# Inhibition of *α*-Glucosidase, Intestinal Glucose Absorption, and Antidiabetic Properties by* Caralluma europaea*

**DOI:** 10.1155/2018/9589472

**Published:** 2018-08-29

**Authors:** Hayat Ouassou, Touda Zahidi, Saliha Bouknana, Mohamed Bouhrim, Hassane Mekhfi, Abderrahim Ziyyat, abdekhaleq Legssyer, Mohamed Aziz, Mohamed Bnouham

**Affiliations:** ^1^Laboratory of Physiology, Genetics and Ethnopharmacology URAC-40, Department of Biology, Faculty of Sciences, University Mohamed I, Oujda, Morocco; ^2^Laboratory of Water, Environment and Sustainable Development, Department of Biology, Faculty of Sciences, University Mohamed I, Oujda, Morocco

## Abstract

Many medicinal plants around the world are used for therapeutic purposes against several diseases, including diabetes mellitus. Due to their composition of natural substances that are effective and do not represent side effects for users, unlike synthetic drugs, in this study, we investigated the inhibitory effect of* Caralluma europaea* (CE) on* α-*glucosidase activity* in vitro*; then the kinetics of the enzyme were studied with increasing concentrations of sucrose in order to determine the inhibition type of the enzyme. In addition, this effect of* Caralluma europaea* (CE) was confirmed* in vivo *using rats as an experimental animal model. Among the five fractions of CE, only the ethyl acetate fraction of* C. europaea* (EACe) induced a significant inhibition of *α*-glucosidase and its inhibition mode was competitive. The* in vivo* studies were conducted on mice and rats using glucose and sucrose as a substrate, respectively, to determine the oral glucose tolerance test (OGTT). The results obtained showed that the EACe and the aqueous extract of* C. europaea* (AECe) have significantly reduced the postprandial hyperglycemia after sucrose and glucose loading in normal and diabetic rats. AECe, also, significantly decreased intestinal glucose absorption,* in situ*. The results obtained showed that* Caralluma europaea* has a significant antihyperglycemic activity, which could be due to the inhibition of *α*-glucosidase activity and enteric absorption of glucose.

## 1. Introduction

Diabetes mellitus (DM) is one of the most serious chronic diseases characterized by a chronic hyperglycemia resulting from defects in insulin secretion and/or insulin action [1]. It can be categorized into two types: type 1 and type 2. Type 1 diabetes mellitus [T1DM] results from an absolute deficiency of insulin, while type 2 diabetes mellitus [T2DM] is due to insulin resistance [2]. WHO has announced an increasing prevalence of diabetes mellitus over the past few decades in different parts of the world [3]. It is estimated that the number of people having diabetes mellitus will increase to 366 million by 2030 [4, 5]. T1DM represents 5%-10% of the total number of diabetes cases worldwide [6]. The management of DM without any side effects is still a challenge to the medical system [7]. Most epidemiological data implicate postprandial hyperglycemia in the development of chronic complications [8]. Control of postprandial plasma glucose level is the treatment aim of diabetes. One therapeutic approach for treating this disease is the inhibition of postprandial hyperglycemia. This is done by the inhibition of intestinal *α*-glucosidases (delaying the process of carbohydrates hydrolysis and absorption of glucose) [7]. Predominantly medicinal plants have been used in Morocco to treat diabetes mellitus due to their traditional acceptability and lesser side effects [9–11].

Numerous ethnopharmacological surveys based on the information from the herbalists and inhabitants classify the selected plants as antidiabetic plants [12]. Within the* Caralluma* genus,* Caralluma tuberculata*,* Caralluma sinaica*,* Caralluma umbellata Haw,* and* Caralluma fimbriata* have all been proven to have antidiabetic properties [13–18]. The plant* Caralluma europaea* (CE) belongs to the family Apocynaceae (subfamily Asclepiadaceae).* Caralluma europaea* is one of Moroccan medicinal plants commonly used in traditional medicine, found in Morocco, Algeria, Egypt, Spain, and Italy [19]. In Moroccan traditional medicine, aerial parts of* C. europaea* are commonly used as a powder or as a juice in the treatment of diabetes mellitus [20]. To the best of our knowledge, no pharmacological work was carried out on* C. europaea*. Hence, the present study was undertaken, mainly, to investigate the antihyperglycemic effects of* C. europaea* by evaluating its effect on *α*-glucosidase. In addition, we monitored its effect on postprandial blood glucose level in normal and diabetic rats.

## 2. Materials and Methods

### 2.1. Chemicals

Hexane (C_6_H_14_) (Sigma Aldrich, Germany), dichloromethane (CH_2_C_l2_) (Sigma Aldrich, Germany), methanol (CH_3_OH) (Sigma Aldrich, Germany), ethyl acetate (C_4_H_8_O_2_) (Sigma Aldrich, France), diethyl ether (Sigma Aldrich, Germany), *α*-glucosidase enzyme (*Saccharomyces cerevisiae*) (Sigma Aldrich, G5003-Sigma, USA), D(+)-glucose (Sigma Aldrich Riedel-de Haen, Seelze), phlorizin hydrate (Sigma Aldrich, USA), Alloxan (ACROS Organics, New Jersey, USA), streptozotocin (Sigma Aldrich, USA), and acarbose (Glucor 50) were obtained from Bayer Schering Pharma (Casablanca, Morocco). Pentobarbital was purchased from CEVA santé animale (La Ballastière, France). Dimethylsulfoxide (DMSO) (C_6_H_6_OS) was obtained from Prolabo (Paris, France), glucose kit was purchased from SGM Italia (Roma, Italy), sodium chloride [NaCl] (Sigma Aldrich, Riedel-de Haen, Denmark), potassium chloride [Kcl] (Sigma Aldrich Riedel-de Haen, Germany), magnesium chloride-6-hydrate [MgCl_2_, 6H_2_O] (Sigma Aldrich Riedel-de Haen, Germany), calcium chloride dihydrate [CaCl_2_, 2H_2_O] (Scharlau Chemie S.A., Spain), sodium hydrogen carbonate [NaHCO_3_] (Farco Chemical Supplies, Puerto Rico), sodium phosphate monobasic 2-hydrate [NaH_2_PO_4_. 2H_2_O] (Panreac, Spain), citric acid (Farco Chemical, Puerto Rico), sucrose was obtained from Prolabo (Rhone-Poulenc Group, European Economic Community, EEC), and glibenclamide was purchased from a local pharmacy (Oujda, Morocco) as Benclamide 5 mg. All chemicals used were of analytical grade.

### 2.2. Plant Identification and Preparation

The fresh stems of* C. europaea* were bought from the herb market in Oujda (Oriental Morocco). It was identified by the botanist Mohamed Fennane (Scientific Institute of Rabat, Morocco). A voucher specimen of the plant material is deposited in Herbarium, Department of Biology, Faculty of Sciences, Oujda, Morocco, with the accession number (HUMPOM 150).

### 2.3. Extraction Method

**Figure 1:**
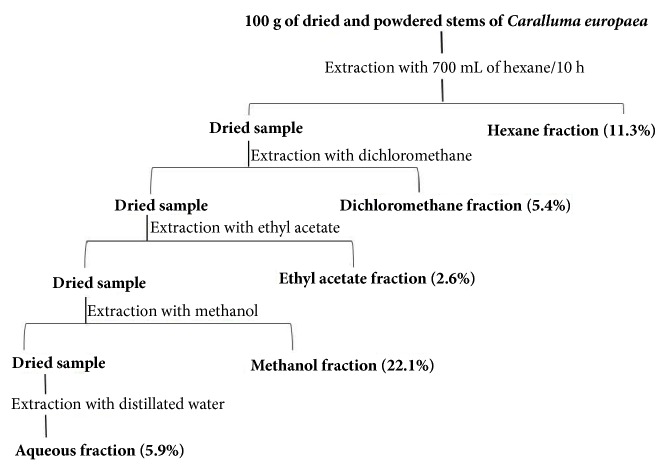
Soxhlet extraction method of stems of* Caralluma europaea* with the yield of each fraction.

The stem samples were washed with water to remove dust and foreign particles. The stems were dried at 45°C. After drying, the stems were ground into powder by using a grinder. The powdered sample (100 g) was extracted with different solvents (700 mL) in soxhlet extractor for 10 hours, followed by evaporation of each solvent in rotary evaporator to give a semisolid residue. The yield of each sample is shown in Figure 1.

### 2.4. Alpha-Glucosidase Inhibition Assay

The *α*-glucosidase, inhibiting the effect of* Caralluma europaea,* was quantified colorimetrically by monitoring the glucose release from sucrose using modified method described by Kim et al. [21, 22]. The assay mixtures contained 0.1 mL of alpha-glucosidase solution (10 IU), 1 mL of phosphate buffer (pH =7.5) and 0.1 mL of sucrose (50 mM) was used as a substrate. Ten and twenty microliters (165 *μ*g/mL and 328 *μ*g/mL) for each sample of* C. europaea *and the same volume of distilled water, 0.3% DMSO, or acarbose (165 *μ*g/mL and 328 *μ*g/mL) as a control and negative and positive controls were used, respectively. The hexane (HCe) and dichloromethane (DCe) fractions were solubilized in DMSO and a buffer solution. Ethyl acetate (EACe), methanol (MCe), and distilled water fractions were solubilized in the buffer solution. The mixture was incubated at 37°C for 20 minutes, and then the reaction was stopped by heating at temperature of 100°C for 5 min in a water bath. The optical density values of the wells were read at 500 nm by spectrophotometry (UV-1800 UV/VIS Spectrophotometer). The concentration of glucose liberated from the reaction was determined by the glucose oxidase method using a commercially available kit (Glucose, SGM Italia). Measurements were carried out in triplicate for each experiment.

The percentage of inhibition was calculated according to the following formula [7]:

(1) Inhibitory  activity%=ODControl−ODTest  sampleODControl×100

### 2.5. Kinetics of *α*-Glucosidase Inhibition by CE

Inhibition mode of EACe against *α*-glucosidase activity was determined with increasing concentrations of sucrose (1-2-3-4-6 mM) as a substrate in the absence or presence of EACe at two different concentrations (500 *μ*g/ml and 1000 *μ*g /ml). Optimal doses were selected on results from the inhibitory activity described above and from preliminary tests of kinetics inhibition of *α*-glucosidase. The data determined by Lineweaver-Burk plot method analysis of the data, which was calculated from the results according to Michaelis-Menten kinetics [23].

### 2.6. Experimental Animals

Swiss albino mice weighing 20-30 g and Wistar rats weighing 130-250 g of each sex were used in the experiments. They were maintained under standard environmental conditions (cycle of 12 h light /12h dark and temperature of 23 ± 2°C) at the Faculty of Sciences, Mohammed I University, Oujda, Morocco. They were kept in cages and fed a standard diet and water* ad libitum*. All animals were cared for in compliance with the Guide for the Care and Use of Laboratory Animals, US National Institutes of Health [24].

### 2.7. Induction of Diabetes

#### 2.7.1. Streptozotocin Induced Hyperglycemia

Diabetes was induced on the basis of protocol described by Wu et al. [25] with minor modifications. Adult rats, fasted for 16 hours, were injected intraperitoneally with 60 mg/kg (body weight) of streptozotocin (STZ) dissolved in fresh and cold phosphate sodium citrate buffer pH= 4.5. Animals with a fasting blood glucose levels more than 1.26 g/L were considered diabetic and selected for the test.

#### 2.7.2. Alloxan Induced Hyperglycemia

Experimental diabetes mellitus was induced on the basis of protocol described by Prince et al. [26] with some modifications. Diabetes was induced by a single intraperitoneal injection (150 mg/kg of body weight) of alloxan monohydrate (Allx monohydrate 98%, ACROS Organics) dissolved in fresh and cold phosphate citrate buffer pH= 4.5. Animals with blood glucose levels more than 1.26 g/L were included in the study.

### 2.8. Acute Oral Toxicity Study

In order to study any possible toxic effect or changes in normal behavior of a single oral administration of* C. europaea *[27], the present study was carried out on 72 albino mice fed normal diet and supplied with tap water. These mice were divided into 12 groups (each of 6 mice). All animals were fasted 16 hours before the experiment. The aqueous extract (AECe) was administered orally, at single doses of 1000, 2000, 3000, 4000, 6000, and 8000 mg/kg body weight, respectively, while the control group received 10 mL/kg of distilled water. The ethyl acetate fraction (EACe) of* C. europaea *was administered orally, at single doses of 100, 300, 500, and 700 mg/kg of body weight. Distilled water was administered to the control group. After administration of these doses, the mortality and general behavior of the animals were observed continuously for 14 days. All surviving animals were sacrificed by overdose of anesthesia by ethylic ether at day 14.

### 2.9. Oral Sucrose Tolerance Test (*OSucTT*) in Normal and Diabetic Rats

To affirm the inhibitory effect of ethyl acetate fraction of* C. europaea* (EACe) on intestinal *α*-glucosidase,* in vivo*, an experimental design described by Ortiz-Andrade et al. [28] was used with some modifications. Rats were deprived of food for 16 h before experimentation with free access to water. The animals were divided into three groups of six animals/group. Groups 1 and 2 were treated with distillated water and EACe by gavage, respectively, at 10 mL/kg and 50 mg/kg. Group 3 was treated with acarbose at 10 mg/kg. 30 minutes after gavage of distillated water, test sample, and acarbose, the animals were orally loaded with sucrose (2 g/kg of body weight). Blood was collected from the tail vein under light anesthesia, at 0, 30, 60, and 120 min. Blood glucose concentration was determined by the glucose oxidase-peroxidase method.

### 2.10. Oral Glucose Tolerance Test (*OGTT*), in Normal and Diabetic Mice

The antihyperglycemic effect of aqueous extract was evaluated as described by chakravarty et al. [29] with some modifications. Animals fasted for 16 h before experimentation but were allowed to free access of water. Normal and diabetic mice were divided into three groups of six mice each. Group 1 (control group) was treated with distilled water (10 mL/kg). Groups 2 and 3 were treated with aqueous extract (AECe) of* C. europaea* and glibenclamide by gavage* (p.o.),* respectively, at 200 mg/kg and 2 mg/kg. All animals were orally loaded with glucose (1g/kg of body weight) 30 minutes after treatments. Blood was collected from the tail tip at 30, 90, 150, and 210 min and centrifuged in a hematocrit centrifuge (Hermle Z 230H, HERMLE, Gosheim, Germany) for 10 min. Serum was separated and blood glucose concentration was determined by the glucose oxidase-peroxidase method (Glucose, SGM Italia).

### 2.11. Intestinal Glucose Absorption,* In Situ*

A protocol is described by Ponz et al. [30] in order to assess the effect of AECeon intestinal absorption of D-glucose in 36 h with free access to water. Fasted normal rats were anaesthetized by intramuscular injection of sodium pentobarbital (50 mg/kg). AECe was added to the perfusion solution (g/L: 7.37 NaCl, 0.2 KCl, 0.065 NaH_2_PO_4_. 2H_2_O, 0.213 MgCl_2_. 6H_2_O, 0.6 NaHCO_3_ and 1.02 CaCl_2_. 2H_2_O, pH=7.5) supplemented with glucose (1 g/L) just before starting the experiment and perfused with a peristaltic pump (Thermo Fisher Scientific Inc., Waltham, MA) at 0.53 mL/min. Three groups of Wistar rats weighing 150-250 g were used: one served as control and received the perfusion solution; one group served as positive control and received the solution with added phlorizin (0.1 mM), a standard inhibitor of D-glucose luminal absorption [31]; and one group received the solution with added 250 mg/kg of AECe. After 60 min, the perfusate was collected from a polyethylene tube at proximal ends and glucose concentration was measured by glucose oxidase-peroxidase method using a commercial kit (Glucose, SGM Italia). The length of the perfused jejunal segment was measured and the intestinal glucose absorption was estimated in mg/10cm/1h.

### 2.12. Statistical Analysis

Data were expressed as mean ± SEM. Statistical analysis and comparison of means were evaluated by one-way analysis of variance (ANOVA). The differences were considered statistically significant at* p *< 0.05.

## 3. Results

### 3.1. Alpha-Glucosidase Inhibitory Activity of CE

**Figure 2:**
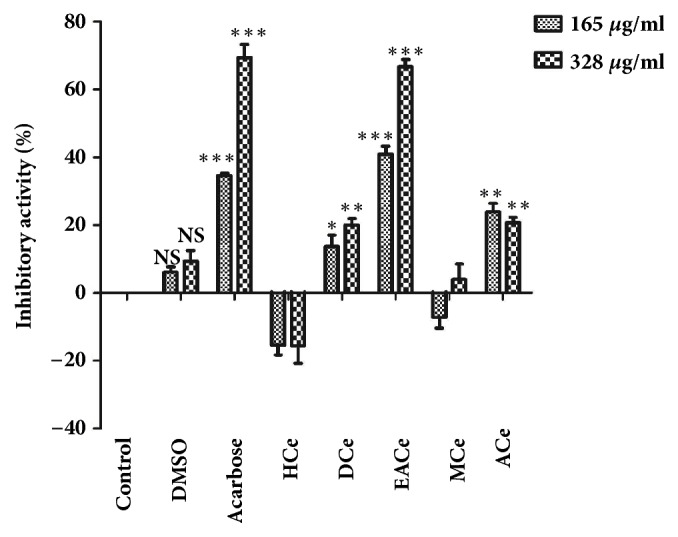
Inhibitory activity of* Caralluma europaea* fractions at two different doses. The essay mixture contained 0.1 mL sucrose (50 mM), 0.1 mL enzyme solution (10 UI), 1 mL of phosphate buffer (50 mM, pH=7.5), and plant fractions at two different concentrations (165 and 328 *μ*g/mL) or acarbose (165 and 328 *μ*g/mL). The reaction mixture was incubated at 37°C for 20 minutes. The amount of liberated glucose was measured by the glucose oxidase method, using a commercial test kit. Values are mean ± SEM (n=3) and are analyzed with one-way ANOVA followed by Dunnett's t-test. Ns = not significant; *p*^*∗*^< 0.05 versus control; *p*^*∗∗*^< 0.01 versus control; *p*^*∗∗∗*^< 0.001 versus control. HCe = hexane fraction of* C. europaea*; DCe = dichloromethane fraction of* C. europaea*; EACe= ethyl acetate fraction of* C. europaea*; MCe= methanol fraction of* C. europaea*; and ACe= aqueous fraction of* C. europaea*.

The results from the inhibitory activity of* Caralluma europaea *show that the DCe, EACe, and ACe were found to significantly (*P<0.01 *and* P<0.001*) inhibit *α*-glucosidase at two doses compared to the control, while HCe and MCe showed no significant effect on the activity of the enzyme. It was observed that EACeat, a dose of 328 *μ*g/mL (66%), has shown the highest inhibitory activity compared to acarbose (69%) (Figure 2).

### 3.2. Kinetic Analysis of Alpha-Glucosidase Inhibition by EACe

**Figure 3:**
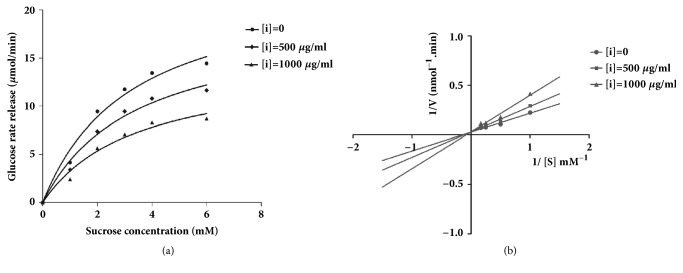
Kinetics of *α*-glucosidase inhibition by ethyl acetate fraction (EACe). (a) Release rate of glucose depending on the concentration of sucrose. (b) Lineweaver-Burk plot of kinetic analysis of *α*-glucosidase inhibition by EACe. *α*-glucosidase was treated with various concentrations of sucrose (1-2-3-4-6 mM) in the absence or presence of EACe at two different doses (500 and 1000 *μ*g/mL). The enzymatic reaction was performed by incubating the mixture at 37°C for 20 minutes. Glucose released was detected colorimetrically by adding a commercial test kit.

Figure 3(a) shows that the rate of glucose release is maximal in the absence of inhibitor. However, the rate of glucose release is lower in the presence of EACe (500 and 1000 *μ*g/mL). The inhibition mode of EACe against *α*-glucosidase activity was analyzed using Lineweaver-Burk plots [32]. Double reciprocal plots of enzyme kinetics demonstrated a competitive inhibition of *α*-glucosidase activity (Figure 3(b)). K_m_ and V_max_ values of EACe against *α*-glucosidase are shown in Table 1.

### 3.3. Acute Oral Toxicity

In the acute toxicity test, aqueous extract (AECe) and ethyl acetate fraction (EACe) of* Caralluma europaea* exhibited no signs of toxicity and no changes in general behavior or other physiological activities of rats. No significant changes were observed in daily food intake in the treated rats as compared to the controls. Both the normal controls and treated rats appeared healthy during the experiment.

### 3.4. Effect of EACe on Oral Sucrose Tolerance in Normal and Diabetic Rats

**Figure 4:**
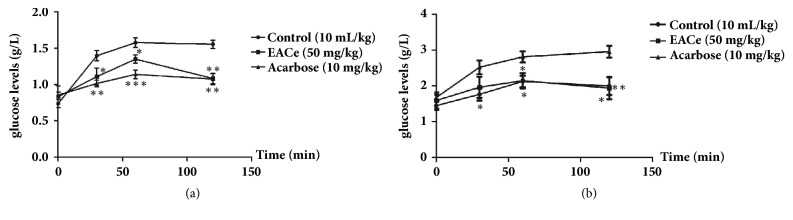
Effect of EACe on serum glucose level after sucrose loading in normal (a) and STZ-diabetic rats (b). The rats which fasted 16 hours received EACe (50 mg/kg), acarbose (10 mg/kg), or only distillated water (control) and 30 minutes after, sucrose was administered (p.o.) at 2 g/kg. Blood samples were collected from the tail tip under anesthesia at 30, 60, and 120 min after sucrose loading. Values are mean ± SEM (n = 6) and are analyzed with one-way ANOVA followed by Dunnett's t-test. *p*^*∗*^< 0.05 versus control; *p*^*∗∗*^< 0.01 versus control; and *p*^*∗∗∗*^< 0.001 versus control.

The results of the oral sucrose (2 g/kg body weight) tolerance test in normal and diabetic rats showed that EACe at dose of 50 mg/kg significantly (*P<0.05* and* P<0.01*) decreased the serum glucose level after sucrose loading, especially at 30, 60, and 120 minutes compared with control group. Acarbose (10 mg/kg) significantly (*P<0.01* and* P<0.001*) reduced the blood glucose level. The oral tolerance test was repeated in STZ-diabetic rats. Similar diminution in blood glucose levels was observed in EACe-treated diabetic group at a dose of 50 mg/kg. Furthermore, acarbose continued to significantly reduce glucose level until the end of the test (Figures 4(a) and 4(b)).

### 3.5. Effect of AECe on Oral Glucose Tolerance Test (OGTT), in Normal and Diabetic Mice

**Figure 5:**
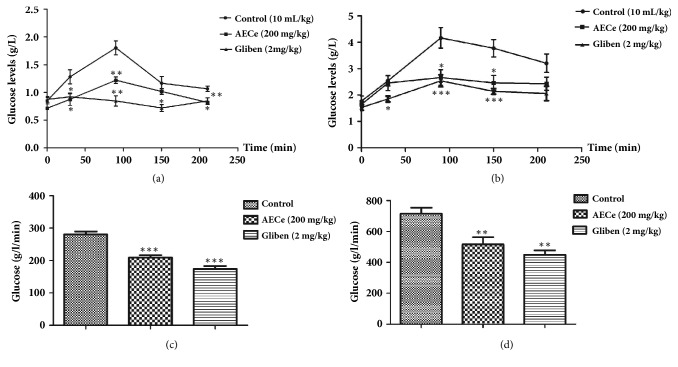
Effect of AECe on serum glucose level after glucose loading in normal (a) and diabetic mice (b). The mice which fasted for 16 h received AECe (200 mg/kg), glibenclamide (2 mg/kg), or only distillated water (control) and 30 minutes after, glucose was administered (*p.o.*) at 1 g/kg. Blood samples were collected from the tail tip under anesthesia at 30, 90, 50, and 210 min after glucose loading. (c) AUC_glucose_ in normal mice and (d) AUC_glucose_ in normal mice. Values represent the mean ± SEM (n = 6) are and analyzed with one-way ANOVA followed by Dunnett's t-test. *p*^*∗*^< 0.05 versus control; *p*^*∗∗*^< 0.01 versus control; and *p*^*∗∗∗*^< 0.001 versus control.

Figure 5 showed that, in control group, the postprandial hyperglycemia level caused by 1 g/kg of glucose loading reached 1.28 g/L 30 min after glucose administration. In 90 mins, it increased to 1.8 g/L and then decreased to reach an average of 1.16 g/L 150 min after loading. However, AECe at dose of 200 mg/kg suppressed significantly the postprandial hyperglycemia level (*P<0.05* and* P<0.01*) compared with the normal control group. Glibenclamide significantly (*p<0.01*) decreased the blood glucose level at 90 min. The area under the curve (AUC_glucose_) of glucose tolerance for the AECe-treated group was significantly lower than that of normal control group. Likewise, AUC values of glibenclamide group were significantly lower than that of control group. In the Allx-induced diabetic mice, oral administration of AECe (200 mg/kg) to Allx-induced diabetic mice significantly (*P<0.01*) reduced serum glucose levels as compared to the diabetic control group, and the area under the curve (AUC_glucose_) of AECe-treated diabetic mice was significantly lower than that of diabetic control group.

### 3.6. Intestinal Glucose Absorption

**Figure 6:**
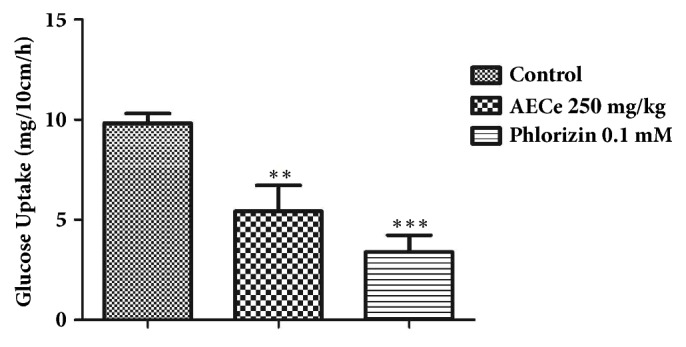
Effect of the aqueous extract of* C. europaea* (AECe) on intestinal absorption in rats. A 10 cm of rat jejunum was perfused with a solution of perfusion containing glucose (1g/L) and the aqueous extract of* C. europaea* at 250 mg/kg. After 60 min, the glucose absorbed was estimated. Values are mean ± SEM (n = 6) and are analyzed with one-way ANOVA followed by Dunnett's t-test. *p*^*∗*^<0.05 versus control, *p*^*∗∗*^<0.01 versus control, and *p*^*∗∗∗*^<0.001 versus control.

As shown in Figure 6, in the absence of test substances, the amount of glucose uptake was 9.82 mg/10cm/h. The presence of AECe (250 mg/kg) significantly (*P<0.01*) reduced glucose absorption rate to 5.41 mg/10cm/h compared to the control. Phlorizin (0.1 mM) decreased significantly (*P<0.001*) the amount of glucose absorbed to 3.38 mg/10cm/h in comparison with the control.

## 4. Discussion

The goal of diabetic patient treatment is to maintain near normal levels of glycemic control, in both the fasting and postprandial states. Many natural resources have been investigated with respect to the suppression of glucose production from carbohydrates in the gut or glucose absorption from the intestine [33]. *α*-amylases catalyze the hydrolysis of *α*-1,4-glucosidic linkages in starch, glycogen, and various oligosaccharides and further degraded to absorbable monosaccharides by *α*-glucosidase, readily available for the intestinal absorption.

One of the therapeutic approaches is to delay the absorption of glucose by carbohydrate hydrolyzing enzymes, *α*-amylases and *α*-glucosidase inhibition, in the digestive tract of humans [34, 35]. Hence, the search for *α*-glucosidase inhibitors from medicinal plants has become a very meaningful task [36]. This work was undertaken to study the effect of* Caralluma europaea* on two pathways of postprandial hyperglycemia. According to our results, EACe showed 66% inhibition of *α*-glucosidase activity* in vitro*. The Lineweaver-Burk plot showed that EACe inhibit the *α*-glucosidase enzyme by competitive binding. We have tested EACe,* in vivo, *in normal and STZ-induced diabetic rats. Oral administration of EACe showed a significant decrease in blood levels in normal and diabetic rats after sucrose loading. These results demonstrated the glucose lowering effect of EACe* in vivo*, possibly due to *α*-glucosidase activity observed* in vitro*. This can be explained by retarding the postprandial glucose levels by the inhibition of intestinal *α*-glucosidase [37]. Similar studies have also reported that the antihyperglycemic activity seems to be more effective* in vivo* than* in vitro*. This effect could be explained by two mechanisms: the inhibition of sucrose degradation by intestinal *α*-glucosidase complex and also the intestinal glucose release blockage via glucose transporter SGLT1 and or/ GLUT2 [37, 38].

In the light of these findings, other experiments are necessary to corroborate and clarify this hypothesis. On the other hand, the effect of AECe on oral glucose tolerance test showed a significant (p < 0.05 and p < 0.01) decrease in fasting blood glucose levels in normal and Allx-induced diabetic mice. The hypoglycemic action of AECe could be related to extrapancreatic and pancreatic secretions [39, 40]. Additionally, we have shown that intestinal glucose absorption was significantly reduced in the presence of EACe or phlorizin. It is well known that phlorizin (glucoside flavonoid) is a specific inhibitor of the sodium-dependent glucose transporter SGLTs. A possible explanation for these results is that AECe exert an inhibitory effect on intestinal glucose transporters, as Phlorizin's principal pharmacological action is to block glucose transportation by inhibition of the sodium-linked glucose transporters (SGLTs) located in the mucosal of the small intestine [41, 42]. However, further experiments will be necessary to corroborate this hypothesis. These results suggest that the antihyperglycemic effect of* Caralluma europaea *is due to the presence of various compounds, which could be responsible for this therapeutic effect. The characteristic phytochemical constituents in Caralluma species are glycosides, flavonoids glycosides, triterpenoids (*β*-sitosterol and lupeol), and saponins [43, 44]. In previous investigations on *α*-glucosidase inhibitors isolated from medicinal plants, some authors suggest that several potential inhibitors belong to flavonoids glycosides class and have the characteristic structural features to inhibit *α*-glucosidase enzyme [45]. On the other hand, *β*-sitosterol induced the uptake of insulin from pancreatic *β*-cells and produced an antihyperglycemic effect [46]. Likewise, lupeol showed to have antihyperglycemic activity [47, 48]. It has been found that bioactive saponins constituents suppress the transfer of glucose from the stomach to the small intestine and inhibit glucose and fluid transport at the brush border membrane [45].

Consequently, we suggest that antihyperglycemic effect of* Caralluma europaea *is produced by the presence of some of these components and/or other phytochemical constituents that can act separately or synergistically. Phytochemical studies are necessary to isolate and identify active constituents responsible for antihyperglycemic effect of this medicinal plant.

## 5. Conclusion

In conclusion, the antihyperglycemic action of* Caralluma europaea* may be partially attributed to the intestinal *α*-glucosidase inhibition as well as to other mechanism pathways which needs to be clarified. Furthermore, phytochemical studies will be required to identify active compounds.

## Figures and Tables

**Table 1:** Kinetic analysis of *α*-glucosidase inhibition by EACe.

Extract concentration (*µ*g/ml)	K_m_ (mM)	V_max_ (*µ*mol/min)
0	7	36.76
500	8.68	33.89
1000	12.39	33.55

## Data Availability

The data used to support the findings of this study are available from the corresponding author upon request.
